# Association of *SLC22A1*, *SLC22A2*, *SLC47A1*, and *SLC47A2* Polymorphisms with Metformin Efficacy in Type 2 Diabetic Patients

**DOI:** 10.3390/biomedicines10102546

**Published:** 2022-10-12

**Authors:** Peixian Chen, Yumin Cao, Shenren Chen, Zhike Liu, Shiyi Chen, Yali Guo

**Affiliations:** 1 Department of Endocrinology and Metabolism, the Second Affiliated Hospital of Shantou University Medical College, Shantou 515000, China; 2 Department of Epidemiology and Biostatistics, School of Public Health, Peking University Health Science Center, Beijing 100191, China

**Keywords:** metformin, single-nucleotide polymorphism, pharmacogenetics, type 2 diabetes mellitus

## Abstract

Response to metformin, first-line therapy for type 2 diabetes mellitus (T2DM), exists interindividual variation. Considering that transporters belonging to the solute carrier (SLC) superfamily are determinants of metformin pharmacokinetics, we evaluated the effects of promoter variants in organic cation transporter 1 (OCT1) (*SLC22A1* rs628031), OCT2 (*SLC22A2* rs316019), multidrug and toxin extrusion protein 1 (MATE1) (*SLC47A1* rs2289669), and MATE2 (*SLC47A2* rs12943590) on the variation in metformin response. The glucose-lowering effects and improvement of insulin resistance of metformin were assessed in newly diagnosed, treatment-naive type 2 diabetic patients of Han nationality in Chaoshan China (*n* = 93) receiving metformin. Fasting plasma glucose (FPG), fasting insulin (FINS), glycated hemoglobin A1 (HbA1_C_), homeostasis model assessment-insulin sensitivity (HOMA-IS), and homeostasis model assessment-insulin resistance (HOMA-IR) were the main metformin efficacy measurements. There were significant correlations between both *SLC47A1* rs2289669 and *SLC47A2* rs12943590 and the efficacy of metformin in individuals with T2DM. In normal weight T2DM patients, significant associations between the AA and GG genotypes of the rs2289669 variant of *SLC47A1* and a greater reduction in FINS and HOMA-IR were detected. A significant correlation was observed between the AG genotype of the rs12943590 polymorphism of *SLC47A2* and a greater reduction in HOMA-IR. Gene–environment interaction analysis showed that in the FINS interaction model, the second-order of dose30_g-*SLC47A2* rs12943590 was statistically significant. The variants of *SLC47A1* rs2289669 and *SLC47A2* rs12943590 could be predictors of insulin resistance in type 2 diabetic patients treated with metformin. The second-order interaction of dose30_g-*SLC47A2* rs12943590 may have a significant effect on FINS in patients with T2DM on metformin treatment. These findings suggest that promoter variants of *SLC47A1* and *SLC47A2* are important determinants of metformin transport and response in type 2 diabetes mellitus.

## 1. Introduction

Metformin is the first-choice oral drug to control blood glucose, which can be used for monotherapy and combination therapy [1,2,3]. It can effectively reduce FPG, postprandial blood glucose (PPG), and HbA1_C_ by inhibiting glycogen production in the liver [4,5,6]. Several genes that can regulate metformin response, such as *SLC22A1* [7,8,9,10], *SLC22A2* [11], *SLC47A1* [12,13], and *SLC47A2* [14], have been identified by previous pharmacogenetic studies. Other genes, such as *ATM* [15,16,17], are involved in regulating metabolic enzymes.

A large number of new T2DM susceptible loci have been found by genome wide association studies in recent years. Several studies have evaluated the relationship between genetic variations, such as *SLC22A1* rs628031, *SLC22A1* rs622342, *ATM* rs11212617, *SLC22A2* rs316019, *SLC47A1* rs2289669, and *SLC47A2* rs12943590, and the pharmacokinetics and clinical consequences of metformin with conflicting results [18,19,20,21].

Previous pharmacogenetic studies found metformin was equally effective among type 2 diabetic patients with a different body mass index (BMI) [22,23,24,25]. There are great individual differences in genetic polymorphism among different populations. Our previous findings that common variants of *ATM* rs11212617 and *SLC22A1* rs622342 may be associated with the effects of metformin treatment on type 2 diabetic patients of Han nationality in Chaoshan China were published in the journal, Pharmacogenetics and Genomics [26].

OCT1 and OCT2, which are encoded by *SLC22A1* and *SLC22A2*, respectively, involve the transfer of endogenous physiological amino compounds [10]. *SLC47A1* and *SLC47A2* genes may mediate the transport and excretion of metformin [27]. Some variants of these genes can affect the glycemic response to metformin. Studies in patients showed that rs628031 and rs316019 in the *SLC22A1* and *SLC22A2* genes, respectively, could exert a significant effect on the distribution and elimination of metformin [28,29]. *SLC47A1* rs2289669 and *SLC47A2* rs12943590 have been shown to influence the reduction of HbA1c in response to metformin treatment [30]. There are studies reporting the allele and genotype frequency of the four common variants (*SLC22A1* rs628031, *SLC22A2* rs316019, *SLC47A1* rs2289669, and *SLC47A2* rs12943590) in other populations; however, so far, there are no data available for the Han nationality in the Chaoshan area of China.

Hence, the current study was mainly aimed to determine the genotype and allele frequency of these four single nucleotide polymorphisms (SNPs), and investigate the relationship between the genetic variants of these four SNPs and the efficacy of metformin in T2DM patients of Chaoshan area Han population.

In addition, type 2 diabetes mellitus is a polygenic genetic disease. It is of great significance to study gene–gene and gene–environment interaction to guide personalized drug use. However, little is known about gene–environment and gene–gene interactions in the efficacy of metformin, such as FPG, HbA1C, FINS, insulin resistance (IR), and β-cell function. Therefore, the purpose of this study was to evaluate whether gene–environment and gene–gene interactions were related to the efficacy of metformin, and explore the predictive function of gene–environment and gene–gene interactions in the individual differences in metformin treatment of patients with type 2 diabetes mellitus.

## 2. Materials and Methods

### 2.1. Study Design and Patient Selection

This study was conducted in the Second Affiliated Hospital of Shantou University Medical College. Subjects must meet the following conditions at the same time to be eligible in this study. First, the diagnosis of diabetes mellitus was based on the World Health Organization (WHO) criterion [25]. Second, patients aged 30–65 years with newly diagnosed T2DM were eligible for the study, including both males and females. Third, although the lifestyle was changed, the appropriate fasting blood glucose level (FPG < 7.0 mmol/l) could not be reached. In addition, they had never received hypoglycemic therapy. Fourth, the participants were from the Chaoshan region of China. Fifth, the BMI of the subjects ranged from 18.5 to 30 kg/m^2^. Sixth, patients were excluded if they had diabetes complications, endocrine disorders, chronic gastrointestinal disease, liver dysfunction, renal insufficiency, heart failure, myocardial infarction, systemic inflammatory disease, blood disease, surgery, malignancies, or corticosteroid treatment. Maternal, nursing women, alcoholics, and people who were allergic to metformin were also excluded.

Prior to participating in this study, all the patients provided a written informed consent. The research proposal was approved by the Ethics Committee of this hospital.

After enrolment, all the patients began to receive metformin treatment for two months. In this study, metformin was produced by Zhongmei Shanghai Shiguibao Pharmaceutical Company, located in Shanghai, China. The initial dose of metformin was determined to be 250 mg twice daily or three times daily. If the FPG was greater than 7.0 mmol/L after one month of treatment, the dose of metformin would be adjusted to 500mg twice or three times daily for the following month.

At the first visit, participants were instructed by a trained physician to complete a questionnaire, which collected information on demographic characteristics, medical history, medication, and lifestyle factors (including sweets, tea drinks, smoking, and alcohol consumption). According to standard protocols, anthropometric parameters, such as height and weight, were measured. The BMI was obtained by dividing the weight (kg) by height squared (m^2^). An overnight (>10 h) fasting blood sample was used to test FPG, HbA1_C_, FINS, liver and renal function, and lipid profile. Diabetes education, including some suggestions on diet and exercise, as well as a brief introduction to T2DM, was provided to all the subjects.

During the two-month treatment period, patients underwent four clinical follow-up visits, respectively, on the 15th, 30th, 45th, and 60th days; and four telephone follow-up visits, respectively, on the 7th, 22nd, 37th, and 52nd days. A follow-up questionnaire on medication compliance and adverse reactions was completed by the same physician at each follow-up. FPG, HbA1_C_, FINS, liver and renal function, and lipid profile were measured at the end of this study, whereas on day 30, FPG was also detected to determine whether the dosage was adjusted or not.

### 2.2. Laboratory Methods

The glucose oxidase method was used to measure plasma glucose. Insulin was determined by radioimmunoassay. The blood lipid, liver function, and kidney function were analyzed by an automatic biochemical analyzer. Genomic deoxyribonucleic acid (DNA) was isolated from peripheral blood leukocytes by protein precipitation according to standard operation procedures. According to the technical specifications, the GenomeLab SNP flow genotyping system (Beckman Coulter Inc., Fullerton, California, United States of America) was used to determine the genotypes of *SLC22A1* rs628031, *SLC22A2* rs316019, *SLC47A1* rs2289669, and *SLC47A2* rs12943590. The insulin resistance index was calculated as the product of fasting plasma glucose (mmol/L) and fasting insulin (µU/mL), divided by 22.5. The insulin sensitivity index was calculated as 1 divided by the product of fasting plasma glucose (mmol/L) and fasting insulin (µU/mL).

### 2.3. Definition of Outcomes

Three kinds of outcomes were used to evaluate the relationship between four SNPs and metformin efficacy:

The first outcome was the decrease in FPG on the 30th day or the 60th day (absolute value of the FPG decrease).
The second outcome hinged on the decrease in HbA1c on the 60th day (absolute value of the HbA1c decrease).
The third outcome was the decrease in FINS or HOMA-IR, or the increase in HOMA-IS on the 60th day (absolute value of the FINS or HOMA-IR reduction, or HOMA-IR increase).

### 2.4. Statistical Analysis

Continuous variables presented as mean and standard deviation (SD) were compared using *t*-test analysis among the groups. Categorical variables of which the frequency distribution was expressed in numbers (proportions) were compared by Pearson χ^2^-test among the groups. When the theoretical frequency was less than 5 or the sample size was less than 40, categorical variables were analyzed using the Fisher exact test. The Pearson χ^2^-test was used to assess the Hardy–Weinberg equilibrium for variants of *SLC22A1* rs628031, *SLC22A2* rs316019, *SLC47A1* rs2289669, and *SLC47A2* rs12943590. Regression analysis and *t*-test analysis were applied in genetic associations. Meanwhile, the interference of potential confounders, such as gender, BMI, FPG, FINS, and HbA1_C_, were adjusted. A generalized multifactor dimensionality reduction (GMDR) was used to analyze the correlation between gene–environment and gene–gene interactions, and various indicators of diabetes, such as FPG, HbA1c, FINS, HOMA-IR, and HOMA-IS. The changes of FPG, HbA1c, FINS, HOMA-IR, and HOMA-IS between the sixtieth day and the first day were expressed as ∆60_FPG_, ∆60_HbA1c_, ∆60_FINS_, ∆60_HOMA-IR_, and ∆60_HOMA-IS_, respectively. The decline of FPG between the thirtieth day and the first day was represented with ∆30_FPG_. Moreover, ∆(60-30)_FPG_ represented the change of FPG between the sixtieth day and the thirtieth day. There were several non-normally distributed variables, including ∆30_FPG_, ∆(60-30)_FPG_, ∆60_FPG_, ∆60_HbA1c_, ∆60_FINS_, ∆60_HOMA-IR_, and ∆60_HOMA-IS_; however, the residual errors of them were approximately normal distribution. All the statistical analyses used the SAS Institute’s statistical software (that is, SAS) for Windows version 9.1 (SAS Institute Inc., Cary, CA, USA). A two-tailed *p* value of less than 0.05 was considered statistically significance in the *t*-test, Pearson χ^2^-test, and GMDR. According to multiple testing, significance thresholds of *p* < 0.0125 or *p* < 0.025 were used, which were related to the number of experimental groups. When the number of experimental groups was two, the significant threshold was 0.025. If the number of experimental groups was three, the significant threshold would be 0.0125.

## 3. Results

In total, 93 patients with type 2 diabetes mellitus were included in this study. Their baseline demographic, anthropometric, biochemical, and genetic characteristics were showed in Table 1. Seven patients were unable to continue the study due to adverse reactions, such as abdominal pain, abdominal distension, and diarrhea. Due to poor compliance, four patients who missed metformin for more than ten days withdrew from the study. A total of 82 T2DM patients completed a two-month treatment with metformin in this study. There were 38 males and 44 females having a mean age of 49.80 ± 12.18 years. The participants had a mean FPG of 10.6 ± 3.2 mmol/L, a mean HbA1c of 8.4 ± 2.1%, and a mean FINS of 12.4 ± 9.3 µU/mL. The SNPs were in Hardy–Weinberg equilibrium (*p* > 0.05). Details on the studies can be found in the Supplementary Material, Table S1.

Considering that some factors, such as sex, age, education, smoking, alcohol drink, tea drink, sweet, and biochemical characteristics, might affect the treatment response, we conducted the following analysis presented in Tables S2 and S3. There were no significant differences among the genotypes of the four SNPs at baseline (*p* > 0.0125, 0.025, respectively). Some suggestions on diet, including alcohol drinks, sweets, and tea drinks, were given to all the patients.

Tables S4–S6 and Table 1 show the relationship between different genotypes and metformin efficacy in the subgroup analysis, which was performed to divide the patients into a normal weight group (BMI ≥ 18.5 and <25 kg/m^2^) and an overweight group (BMI ≥ 25 and <30 kg/m^2^) according to BMI. There were no significant differences in the ∆30_FPG_, ∆60_FPG_, ∆(60-30)_FPG_, ∆60_HbA1c_, ∆60_FINS_, ∆60_HOMA-IR_, and ∆60_HOMA-IS_ among the different genotype groups of *SLC22A1* rs628031, *SLC22A2* rs316019, and *SLC47A2* rs12943590 (*p* > 0.0125, 0.025, respectively). For *SLC47A1* rs2289669, the reduction in ∆60_FINS_ and ∆60_HOMA-IR_ after a two-month treatment with metformin was significantly different in the normal weight group (*p* = 0.0086, 0.0044, respectively). The results showed that there were no differences in the others among the genotypes of *SLC47A1* rs2289669 (*p* > 0.025).

The association between the genotypes of these four SNPs and the efficacy of metformin was researched by regression analysis, which was adjusted for several variables, including sex, age, BMI, metformin dosage, education, tea drink, smoking, and sweets. There were no significant differences in ∆60_FINS_ and ∆60_HOMA-IR_ among the different genotypes of *SLC22A1* rs628031, *SLC22A2* rs316019, and *SLC47A1* rs2289669 (*p* > 0.0125, 0.025, respectively). Compared with the GG and AA genotypes, patients with the AG genotype of *SLC47A2* rs12943590 had a greater reduction in ∆60_HOMA-IR_ (*p* = 0.00748). No significant difference was observed in ∆60_FINS_ among the different genotypes of *SLC47A2* rs12943590 (*p* > 0.0125). See more details in Table 2.

GMDR was used to analyze the correlation between the gene–environment interaction and FINS in Table 3. Gene–environment interaction analysis showed that in the FINS interaction model, the second-order of the dosage of metformin on day 30 (dose30_g)-*SLC47A2* rs12943590 was statistically significant (*p* = 0.0107). The balance accuracy and cross-validation consistency were 0.7167 and 5/10, respectively. However, there was no difference in the four-order of dose30_g, *SLC22A1* rs628031, *SLC22A2* rs316019, and *SLC47A1* rs2289669 (*p* = 0.206).

## 4. Discussion

Genetic determinants and environmental factors play an important role in the development of type 2 diabetes, which is a complex metabolic disease [31]. Fewer than two-thirds of T2DM patients achieved appropriate control of FPG [32]. At present, metformin has been recommended as the first choice of treatment drug for T2DM by multinational guidelines. In this study, we studied the correlation between *SLC22A1*, *SLC22A2*, *SLC47A1*, and *SLC47A2* variants and the risk of T2DM in Han Chinese from Chaoshan, China; and explored the effect of genotype on FPG, HbA1c, FINS, HOMA-IR, and HOMA-IS in T2DM patients receiving metformin treatment.

In this prospective cohort study, we investigated the relationship between *SLC22A1* rs628031, *SLC22A2* rs316019, *SLC47A1* rs2289669, and *SLC47A2* rs12943590, and metformin efficacy in T2DM patients. A country-specific point has been established for Asian populations (such as Chinese), and it is recommended that the cutoff point of overweight is 24 kg/m^2^ [33]. Nevertheless, this study adopted the World Health Organization criteria to define normal weight as a BMI ≥ 18.5 and <25 kg/m^2^ and overweight as a BMI ≥ 25 and <30 kg/m^2^, which made it easy to compare studies on different populations [34].

The MATE 1 protein, which is encoded by the *SLC47A1* gene, affects the excretion of metformin into the bile and urine, thereby affecting metformin efficacy [27,35]. In this study, patients were from the Chaoshan area of China and the frequencies of the genotype of *SLC47A1* rs2289669 were 0.061, 0.793, and 0.146 for AA, AG, and GG, respectively. Another study conducted in the Han population in southeast China, including 110 patients treated with metformin for 90 days, reported that the frequencies of the *SLC47A1* rs2289669 genotype in AA, AG, and GG were 0.227, 0.557, and 0.216, respectively [12]. The reason the gene frequency distribution was different may be the regional difference, which may lead to different research results.

To the best of our knowledge, two other studies carried out in a Scottish population and a Chinese population separately reported a positive result, which was that they found a significant effect of *SLC47A1* rs2289669 on the efficacy of metformin. In Scottish individuals, a study involving 148 T2DM patients who received metformin treatment for six months found that *SLC47A1* rs2289669 had a significant genotype-specific effect on the response to metformin, and HbA1c in patients with the homozygous *SLC47A1* rs2289669 A-allele had a significantly lower reduction [8]. The other study showed that among 180 newly diagnosed type 2 diabetic patients with the *SLC47A1* rs2289669 AA genotype, the change of HbA1c level was significantly different [21]. However, no significant difference was found in the HbA1_C_ reduction among the different genotype groups of *SLC47A1* rs2289669 in the study. The reason for the two different results may be related to the differences in gene frequency distribution between regions. Compared with carriers of the AG genotype, normal weight T2DM patients with the *SLC47A1* rs2289669 AA or GG genotype had a better effect in lowering FINS and HOMA-IR after a two-month metformin monotherapy. However, regression analysis showed that there was no significant difference in FINS and HOMA-IR between patients with the *SLC47A1* rs2289669 AG genotype and those with the AA/GG genotype, which showed that this SNP was not associated with metformin efficacy in 82 patients.

The MATE2 transporter is encoded by the *SLC47A2* gene [36]. *SLC47A2* rs12943590, which is one of the most relevant clinical gene variants in the *SLC47A2* gene, affects metformin depuration [37]. Using the statistical method of regression analysis, our study showed that *SLC47A2* rs12943590 played a role in the interindividual variability of metformin efficacy. In the present study, we showed that the AG genotype of *SLC47A2* rs12943590 correlated with a significant reduction in HOMA-IR, whereas the genotypes of *SLC47A2* rs12943590 were not associated with a significant reduction in HbA1c. Significant associations between the AG genotype of *SLC47A2* rs12943590 and a greater reduction of HbA1c were detected, whereas no significant effect of this polymorphism on HOMA-IR was found [30]. Our results were inconsistent with the research conclusions of Phani N.M et al. [30], which may be related to individual differences in gene frequency distribution.

If racial genetic heterogeneity is excluded, the effect of gene–gene and gene–environment on the pharmacokinetic and pharmacodynamic efficacy of metformin may be an important factor in the difference. Previous studies have shown that *MATE1* rs2252281-*SLC22A2* rs316019 and *SLC22A1* rs622342-*SLC47A1* rs2289669 had significant effects on the pharmacokinetic and pharmacodynamic efficacy of metformin, respectively [38,39]. However, the interaction between *SLC22A2* rs316019 and *SLC47A1* rs2289669 in this study did not enter the model, which suggested that the interaction between *SLC22A2* rs316019 and *SLC47A1* rs2289669 did not significantly affect insulin resistance in patients with type 2 diabetic patients receiving metformin treatment. Our study found that the second-order interaction of dose30_g-*SLC47A2* rs12943590 might have a significant effect on FINS in patients with type 2 diabetes taking metformin; however, other interaction models were not statistically significant (*p* > 0.05).

The results of this study showed that *SLC47A2* rs12943590 predicted insulin resistance improvement in patients with type 2 diabetes mellitus treated with metformin, which may be helpful to guide the clinical use of metformin in the treatment of type 2 diabetes mellitus to some extent. Therefore, for type 2 diabetic patients with the *SLC47A2* rs12943590 AG genotype, metformin may be an appropriate choice.

The present study had some limitations. First, compared with the previous study mentioned above [12], our study lasted for two months, which was not enough to observe the long-term effects of these four SNPs on the therapeutic efficacy of metformin. Our results could only show the preliminary effect, which remains to be verified by further study and longer follow-up time. Second, the participants in the present study were newly diagnosed, treatment-naive type 2 diabetic subjects. Therefore, whether our findings could be extended to patients with long-term T2DM needs further testing. Third, the patients in this study came from the Chaoshan region of China; hence, whether our findings were applicable to patients from other regions remains to be tested. Although there were limitations, metformin monotherapy and prospective design in previous studies could attenuate the influence of confounding factors, such as information bias and concomitant medication.

## 5. Conclusions

To sum up, we found that in a Chaoshan population with newly diagnosed T2DM, rs2289669 of the *SLC47A1* gene and rs12943590 of the *SLC47A2* gene polymorphism might affect the risk of T2DM by changing HOMA-IR parameters and other factors. Our data indicated that the AA or GG genotype of *SLC47A1* rs2289669 and the AG genotype of *SLC47A2* rs12943590 might affect HOMA-IR in patients with type 2 diabetes. No significant effects of both *SLC22A1* rs628031 and *SLC22A2* rs316019 against glycemic response, FINS, HOMA-IR, and HOMA-IS were detected in our study. In addition, dose30_g-*SLC47A2* rs12943590 could be a risk factor for HOMA-IR in T2DM.

## Figures and Tables

**Table 1:** Genotypes of *SLC47A1* rs2289669 on the effect of metformin efficacy in subgroup analysis.

Variables ^a^	Normal Weight Group				Overweight Group			
	AA/GG	AG	F Value	*p* Value	AA/GG	AG	F Value	*p* Value
∆30_FPG_	−0.3 ± 0.2,(8)	−0.2 ± 0.2,(27)	1.87	0.1805	−0.2 ± 0.2,(9)	−0.3 ± 0.2,(35)	1.08	0.3051
∆60_FPG_	−0.4 ± 0.2,(8)	−0.3 ± 0.3,(27)	0.73	0.3990	−0.4 ± 0.2,(9)	−0.4 ± 0.2,(35)	0.42	0.5200
∆(60–30)_FPG_	−0.035 ± 0.126,(8)	−0.057 ± 0.159,(27)	1.33	0.2559	−0.145 ± 0.146,(9)	−0.122 + 0.175,(35)	0.13	0.7155
∆60_HbA1c_	−0.113 ± 0.182,(8)	−0.149 ± 0.208,(29)	0.20	0.6585	−0.222 ± 0.143,(9)	−0.218 ± 0.159,(36)	0.01	0.9418
∆60_FINS_	−0.555 ± 0.258,(6)	−0.010 ± 0.456,(27)	7.87	0.0086	−0.129 ± 0.500,(8)	−0.035 ± 0.534,(35)	0.21	0.6518
∆60_HOMA-IR_	−0.923 ± 0.436,(6)	−0.250 ± 0.494,(27)	9.44	0.0044	−0.488 ± 0.487,(8)	−0.442 ± 0.516,(35)	0.05	0.8202
∆60_HOMA-IS_	0.141 ± 0.294,(6)	0.460 ± 0.815,(27)	0.87	0.3574	0.496 ± 0.631,(8)	0.679 ± 0.720,(35)	0.43	0.5139

^a^ Continuous variables were expressed as mean ± SD, (N) and analyzed using a *t*-test. Genetic associations were tested using regression analysis.

**Table 2:** Genotypes of four SNPs on the effect of metformin efficacy in regression analysis.

Variables ^a^	Genotype	Mean ± SD (*n*)	BETA		BETA	
			Crude	*p* Value	Adjusted	*p* Value
∆60_FINS_	*SLC22A1* rs628031					
	GG	−0.178 ± 0.430 (44)	0.000		0.000	
	GA/AA	0.061 ± 0.561 (32)	0.238	0.03364	0.228	0.03300
	*SLC22A2* rs316019					
	AC	−0.170 ± 0.456 (35)	0.000		0.000	
	CC	0.002 ± 0.527 (41)	0.172	0.12611	0.228	0.03371
	*SLC47A1* rs2289669					
	AA/GG	−0.312 ± 0.456 (14)	0.000		0.000	
	AG	−0.024 ± 0.498 (62)	0.287	0.04491	0.248	0.05860
	*SLC47A2* rs12943590					
	GG	0.090 ± 0.475 (27)	0.000		0.000	
	AG	−0.190 ± 0.461 (37)	−0.280	0.02129	−0.273	0.01884
	AA	−0.105 ± 0.603 (12)	−0.195	0.24290	−0.242	0.12332
∆60_HOMA-IR_	*SLC22A1* rs628031					
	GG	−0.448 ± 0.461 (44)	0.000		0.000	
	GA/AA	−0.373 ± 0.600 (32)	0.075	0.53045	0.083	0.47461
	*SLC22A2* rs316019					
	AC	−0.511 ± 0.475 (35)	0.000		0.000	
	CC	−0.337 ± 0.551 (41)	0.174	0.13896	0.202	0.07673
	*SLC47A1* rs2289669					
	AA/GG	−0.675 ± 0.501 (14)	0.000		0.000	
	AG	−0.359 ± 0.512 (62)	0.316	0.03378	0.274	0.04756
	*SLC47A2* rs12943590					
	GG	−0.208 ± 0.482 (27)	0.000		0.000	
	AG	−0.543 ± 0.472 (37)	−0.335	0.00730	−0.323	0.00748
	AA	−0.499 ± 0.636 (12)	−0.291	0.08887	−0.364	0.02586

^a^ Continuous variables were expressed as mean ± SD, (N) and analyzed using a *t*-test. Genetic associations were tested using regression analysis. Several variables were adjusted, including sex, age, BMI, metformin dosage, education, tea drink, smoking, and sweets.

**Table 3:** Gene–environment interaction model for fasting insulin by GMDR method.

Model	Test Accuracy	The *p* Value of GMDR	CVC	*p* Value
dose30_g, *SLC47A2* rs12943590	0.7167	9 (*p* = 0.0107)	5	0.007
dose30_g, *SLC22A1* rs628031, *SLC22A2* rs316019, *SLC47A1* rs2289669	0.5983	7 (*p* = 0.1719)	8	0.206

The correlation between gene–environment interactions and various indicators of diabetes were tested using GMDR. CVC, cross-validation consistency.

## Data Availability

Data are available on request due to privacy or ethics.

## Supplementary Materials

The following supporting information can be downloaded at: https://www.mdpi.com/article/10.3390/biomedicines10102546/s1. Table S1: Baseline characteristics of the participants in this study; Table S2: Comparison of baseline characteristics of different genotypes of *SLC22A1* rs628031 and *SLC22A2* rs316019;.Table S3: Comparison of baseline characteristics of different genotypes of *SLC47A1* rs2289669 and *SLC47A2* rs12943590; Table S4: Genotypes of *SLC22A1* rs628031 on the effect of metformin efficacy in subgroup analysis; Table S5: Genotypes of *SLC22A2* rs316019 on the effect of metformin efficacy in subgroup analysis; Table S6: Genotypes of *SLC47A2* rs12943590 on the effect of metformin efficacy in subgroup analysis.
