# Supplementary material for: Association of SLC22A1, SLC22A2, SLC47A1, and SLC47A2 Polymorphisms with Metformin Efficacy in Type 2 Diabetic Patients
Source: Biomedicines. 2022 Oct 12;10(10):2546. doi: 10.3390/biomedicines10102546 (PMC9599747; doi:10.3390/biomedicines10102546)
Supplement: Supplementary file 1 [file biomedicines-10-02546-s001.zip › biomedicines-1874775-supplementary.pdf]

Table S1. Baseline characteristics of the participants in this study.

Variables	Whole Cohort (*n* = 82)
Male	38 (46.3%)
Age(years)	49.80 ± 12.18
BMI (kg/m²)	24.34 ± 3.62
FPG (mmol/L)	10.6 ± 3.2
HbA1c (%)	8.4 ± 2.1
FINS (μU/mL)	12.4 ± 9.3
SLC22A1 rs628031	
GG	49 (59.8%)
GA	32 (39.0%)
AA	1 (1.2%)
SLC22A2 rs316019	
AC	36(43.9%)
CC	46(56.1%)
SLC47A1 rs2289669	
AA	5(6.1%)
AG	65 (79.3%)
GG	12 (14.6%)
SLC47A2 rs12943590	
GG	29(35.4%)
AG	39(47.6%)
AA	14(17.1%)

Continuous variables were expressed as mean ± SD and analyzed using t-test. Categorical variables were expressed as *n* (%) and compared using the Pearson χ^2-test.

Table S2. Comparison of baseline characteristics of different genotypes of *SLC22A1* rs628031 and *SLC22A2* rs316019.

Baseline Characteristics	Groups	*SLC22A1* rs628031			*SLC22A2* rs316019		
		GG	GA/AA	*p* Value	AC	CC	*p* value
Sex				0.749			0.557
	Male	22	16		18	20	
	Female	27	17		18	26	
Age a	_	51.45 ± 10.88	48.48 ± 10.76	0.295	52.25 ± 10.36	48.98 ± 11.09	0.176
Educational level				0.887			0.209
	Level A	32	21		26	27	
	Level B	13	9		6	16	
	Level C	3	3		3	3	
Smoking				0.709			0.053
	Yes	13	10		14	9	
	None	36	23		7	22	
Alcohol drink				0.025			0.588
	Yes	1	5		2	4	
	None	48	28		34	42	
Tea drink				0.281			0.740
	Yes	30	24		23	31	
	None	19	9		13	15	
Sweet				0.073			0.664

	Yes	23	9		15	21	
	None	26	24		17	29	
TC(mmol/L)	—	5.23 ± 1.84	5.58 ± 1.48	0.368	5.23 ± 2.17	5.48 ± 1.24	0.539
TG(mmol/L)	—	2.68 ± 2.08	3.02 ± 2.01	0.469	2.79 ± 2.15	2.84 ± 1.98	0.911
HDL(mmol/L)	—	1.29 ± 0.42	1.20 ± 0.32	0.299	1.28 ± 0.39	1.23 ± 0.38	0.615
LDL(mmol/L)	—	3.26 ± 0.93	3.26 ± 0.83	0.983	3.42 ± 0.81	3.13 ± 0.93	0.148
ALT(U/L)	—	36.56 ± 21.21	37.76 ± 24.32	0.815	36.97 ± 24.81	37.11 ± 20.53	0.978
AST(U/L)	—	29.00 ± 14.79	28.36 ± 16.33	0.856	30.28 ± 15.80	27.51 ± 15.03	0.423
Cr(μmol/L)	—	72.54 ± 16.96	73.61 ± 22.25	0.808	74.84 ± 16.65	71.43 ± 21.00	0.431
Bun(mmol/L)	—	5.02 ± 1.38	5.38 ± 1.42	0.273	5.17 ± 1.54	5.15 ± 1.29	0.950

Continuous variables were expressed as mean ± SD and analyzed using t-test. Categorical variables were expressed as N (%) and compared using the Pearson χ^2-test. When the sample size was less than 40 or the theoretical frequency was less than 5, Fisher exact test was used. Genetic associations were tested using a t-test. Level A, Junior secondary and below. Level B, High School / Technical Secondary School. Level C, University and above.

Table S3. Comparison of baseline characteristics of different genotypes of *SLC47A1* rs2289669 and *SLC47A2* rs12943590.

Baseline Characteristics	Groups	***SLC47A1* rs2289669**			***SLC47A2* rs12943590**			
		**AA/GG**	**AG**	***p* value**	**GG**	**AG**	**AA**	***p* value**
Sex				0.631				0.118
	Male	7	31		12	16	10	
	Female	10	34		17	23	4	
Age a	—	52.29 ± 10.37	49.92 ± 10.97	0.425	48.10 ± 9.42	52.92 ± 11.68	48.21 ± 10.26	0.136
Educational level				0.350				0.464
	Level A	13	40		12	26	15	
	Level B	4	18		1	13	8	
	Level C	0	6		1	3	2	
Smoking				0.888				0.027
	Yes	5	18		7	8	8	
	None	12	47		22	31	6	
Alcohol drink				0.193				0.438
	Yes	0	6		1	3	2	
	None	17	59		28	36	12	
Tea drink								0.521
	Yes	11	43	0.911	17	28	9	
	None	6	22		12	11	5	
Sweet				0.060				0.263
	Yes	10	22		11	18	3	
	None	7	43		18	21	11	
TC(mmol/L)	—	4.77 ± 2.12	5.53 ± 1.55	0.103	5.34 ± 1.77	5.35 ± 1.75	5.49 ± 1.50	0.960
TG(mmol/L)	—	3.08 ± 2.09	2.75 ± 2.05	0.559	2.64 ± 1.98	2.69 ± 1.92	3.51 ± 2.51	0.377
HDL(mmol/L)	—	1.37 ± 0.40	1.22 ± 0.38	0.169	1.22 ± 0.37	1.33 ± 0.42	1.10 ± 0.20	0.150
LDL(mmol/L)	—	3.41 ± 0.95	3.22 ± 0.87	0.446	3.47 ± 1.06	3.11 ± 0.72	3.25 ± 0.92	0.271
ALT(U/l)	—	40.50 ± 26.48	36.20 ± 21.41	0.495	36.35 ± 26.25	36.18 ± 20.35	40.86 ± 20.08	0.787
AST(U/l)	—	34.69 ± 17.83	27.28 ± 14.45	0.084	28.48 ± 19.16	27.68 ± 11.68	32.14 ± 15.95	0.651
Cr(μmol/l)	—	73.29 ± 17.43	72.88 ±	0.941	67.43 ±	73.75 ± 16.22	81.90 ± 23.87	0.063

			19.65		19.00			
Bun(mmol/l)	—	5.15 ± 1.07	5.17 ± 1.48	0.973	4.73 ± 1.32	5.35 ± 1.24	5.57 ± 1.88	0.114

Continuous variables were expressed as mean ± SD and analyzed using t-test. Categorical variables were expressed as N (%) and compared using the Pearson χ^2-test. When the sample size was less than 40 or the theoretical frequency was less than 5, Fisher exact test was used. Genetic associations were tested using a t-test. Level A, Junior secondary and below. Level B, High School / Technical Secondary School. Level C, University and above.

Table S4. Genotypes of *SLC22A1* rs628031 on the effect of metformin efficacy in subgroup analysis.

Variables [a]	Normal Weight Group				Overweight Group			
	GG	GA/AA	*F* Value	*p* Value	GG	GA/AA	*F* Value	*p* Value
$\triangle 30_{\text{FPG}}$	−0.2 ± 0.2,(24)	−0.3 ± 0.2 ,(11)	0.54	0.4690	−0.2 ± 0.2 ,(23)	−0.3 ± 0.2,(21)	3.93	0.0541
$\triangle 60_{\text{FPG}}$	−0.2 ± 0.3,(24)	−0.4 ± 0.2,(11)	2.47	0.1256	−0.4 ± 0.2,(23)	−0.5 ± 0.2,(21)	2.02	0.1630
$\triangle(60{-}30)_{\text{FPG}}$	−0.025 ± 0.151,(24)	−0.112 ± 0.137,(11)	4.84	0.0344	−0.137 ± 0.136,(23)	−0.115 ± 0.201,(21)	0.18	0.6723
$\triangle 60_{\text{HbA1c}}$	−0.146 ± 0.217,(25)	−0.131 ± 0.170,(12)	0.05	0.8300	−0.194 ± 0.151,(24)	−0.246 ± 0.157,(21)	1.30	0.2604
$\triangle 60_{\text{FINS}}$	−0.192 ± 0.390,(22)	0.055 ± 0.595,(11)	2.06	0.1615	−0.163 ± 0.474,(22)	0.063 ± 0.558,(21)	2.07	0.1577
$\triangle 60_{\text{HOMA-IR}}$	−0.393 ± 0.478,(22)	−0.331 ± 0.686,(11)	0.09	0.7641	−0.504 ± 0.446,(22)	−0.395 ± 0.567,(21)	0.49	0.4876
$\triangle 60_{\text{HOMA-IS}}$	0.237 ± 0.793,(22)	0.731 ± 0.565,(11)	3.38	0.0755	0.440 ± 0.637,(22)	0.859 ± 0.715,(21)	4.14	0.0483

[a] Continuous variables were expressed as mean ± SD, (N) and analyzed using t-test. Non-normally distributed variables, such as FINS, HOMA-IR, and HOMA-IS, were converted to normally distributed variables and analyzed. Genetic associations were tested using regression analysis.

Table S5. Genotypes of *SLC22A2* rs316019 on the effect of metformin efficacy in subgroup analysis.

Variables [a]	Normal Weight Group				Overweight Group			
	AC	CC	*F* Value	*p* Value	AC	CC	*F* Value	*p* Value
$\triangle 30_{\text{FPG}}$	−0.3 ± 0.2,(15)	−0.2 ± 0.2,(20)	0.21	0.6472	−0.3 ± 0.2 ,(21)	−0.3 ± 0.2,(23)	1.03	0.3164
$\triangle 60_{\text{FPG}}$	−0.3 ± 0.3,(15)	−0.3 ± 0.2,(20)	0.89	0.3532	−0.4 ± 0.2,(21)	−0.5 ± 0.2,(23)	2.35	0.1327
$\triangle(60{-}30)_{\text{FPG}}$	−0.080 ± 0.140,(15)	−0.031 ± 0.158,(20)	1.45	0.2371	−0.106 ± 0.139,(21)	−0.145 ± 0.193,(23)	0.59	0.4464
$\triangle 60_{\text{HbA1c}}$	−0.162 ± 0.207,(15)	−0.127 ± 0.200,(22)	0.20	0.6585	−0.215 ± 0.153,(21)	−0.222 ± 0.158,(24)	0.02	0.8788
$\triangle 60_{\text{FINS}}$	−0.140 ± 0.499,(15)	−0.083 ± 0.465,(18)	0.12	0.7357	−0.192 ± 0.433,(20)	0.069 ± 0.573,(23)	2.78	0.1032
$\triangle 60_{\text{HOMA-IR}}$	−0.477 ± 0.558,(15)	−0.285 ± 0.536,(18)	1.00	0.3241	−0.536 ± 0.417,(20)	−0.377 ± 0.571,(23)	1.06	0.3087
$\triangle 60_{\text{HOMA-IS}}$	0.588 ± 0.935,(15)	0.247 ± 0.543,(18)	1.71	0.2009	0.403 ± 0.578,(20)	0.854 ± 0.742,(23)	4.83	0.0336

[a] Continuous variables were expressed as mean ± SD, (N) and analyzed using t-test. Non-normally distributed variables, such as FINS, HOMA-IR, and HOMA-IS, were converted to normally distributed variables and analyzed. Genetic associations were tested using regression analysis.

Table S6. Genotypes of *SLC47A2* rs12943590 on the effect of metformin efficacy in subgroup analysis.

Variables [a]	Normal Weight Group					Overweight Group				
	GG	AG	AA	*F* Value	*p* Value	GG	AG	AA	*F* Value	*p* Value
△30_{FPG}	−0.2 ± 0.1,(12)	−0.3 ± 0.2,(15)	−0.3 ± 0.2,(8)	2.12	0.1371	−0.2 ± 0.1,(15)	−0.3 ± 0.2,(23)	−0.3 ± 0.2,(6)	0.64	0.5320
△60_{FPG}	−0.2 ± 0.2,(12)	−0.3 ± 0.2,(15)	−0.4 ± 0.4,(8)	0.49	0.6189	−0.3 ± 0.2,(15)	−0.4 ± 0.2,(23)	−0.5 ± 0.2,(6)	1.20	0.3105
△$(60–30)_{FPG}$	−0.086 ± 0.117,(12)	−0.040 ± 0.138,(15)	−0.024 ± 0.218,(8)	1.46	0.2450	−0.112 ± 0.149,(15)	−0.122 ± 0.188,(23)	−0.181 ± 0.151,(6)	0.36	0.6983
△60_{HbA1c}	−0.065 ± 0.135,(13)	−0.165 ± 0.151,(16)	−0.219 ± 0.329,(8)	1.73	0.1931	−0.221 ± 0.182,(16)	−0.214 ± 0.149,(23)	−0.230 ± 0.112,(6)	0.03	0.9714
△60_{FINS}	0.115 ± 0.464,(11)	−0.185 ± 0.431,(16)	−0.319 ± 0.519(6)	2.17	0.1320	0.072 ± 0.496,(16)	−0.194 ± 0.494,(21)	0.110 ± 0.648,(6)	1.56	0.2237
△$60_{HOMA-IR}$	−0.125 ± 0.444,(11)	−0.456 ± 0.514,(16)	−0.603 ± 0.704,(6)	1.97	0.1568	−0.265 ± 0.513,(16)	−0.609 ± 0.439,(21)	−0.395 ± 0.607,(6)	2.28	0.1158
△$60_{HOMA-IS}$	0.517 ± 0.580,(11)	0.278 ± 0.603,(16)	0.521 ± 1.334,(6)	0.40	0.6709	0.693 ± 0.606,(16)	0.498 ± 0.722,(21)	1.030 ± 0.815,(6)	1.44	0.2500

[a] Continuous variables were expressed as mean ± SD, (N) and analyzed using t-test. Non-normally distributed variables, such as FINS, HOMA-IR, and HOMA-IS, were converted to normally distributed variables and analyzed. Genetic associations were tested using regression analysis.
